# Short-term recovery after NovaSure® endometrial ablation: a prospective cohort study

**DOI:** 10.52054/FVVO.14.4.042

**Published:** 2023-01-27

**Authors:** I.M.A. Reinders, M.R.D. van de Kar, P.M.A.J. Geomini, J.C. Leemans, J.W.M. Maas, M.Y. Bongers

**Affiliations:** Department of Obstetrics and Gynaecology, Viecuri Medical Centre, Venlo, The Netherlands; Department of Cardiology, Catharina Hospital, Eindhoven, The Netherlands; Department of Obstetrics and Gynaecology MUMC+ and Grow- school of Oncology and Reproduction, Maastricht University, 6229 ER, Maastricht, The Netherlands; Department of Obstetrics and Gynaecology, Máxima Medical Centre, 5504 DB, Veldhoven, The Netherlands

**Keywords:** NovaSure®, endometrial ablation, recovery, heavy menstrual bleeding, outpatient

## Abstract

**Background:**

Endometrial ablation is a frequently performed treatment for heavy menstrual bleeding, but detailed information about recovery to help inform patients is lacking.

**Objective:**

To gain more insight into the short-term recovery after NovaSure® endometrial ablation, with the goal of improving preprocedural counselling.

**Materials and Methods:**

A total of 61 women who underwent endometrial ablation between March 2019 and November 2021 in a teaching hospital in the Netherlands were included in this prospective cohort study.

**Main outcome measures:**

Short-term recovery was investigated through questionnaires in the first week after the procedure. The primary outcome was the Recovery Index (RI-10). Secondary outcomes included health-related quality of life (EQ-5D-5L), pain intensity, use of analgesics, nausea, vaginal discharge, capability of performing activities (domestic chores, sports, work), self-rated health (EQ-VAS) and the feeling of full recovery.

**Results:**

A total of 33 women underwent the procedure under local anaesthesia and 28 women under procedural sedation. The RI-10 increased in the first week; median scores on day one, two and seven were 34 (IQR 28.5-41.5), 38.5 (IQR 31-47), and 42 (IQR 37.5-48), respectively. The median time for full recovery was five days. However, 23% of all women were not fully recovered within seven days. Women needed a median time of two days for returning to their work and 5.5 days for sporting activities. There were no differences in recovery between both anaesthesia techniques.

**Conclusions:**

Women undergoing endometrial ablation can be informed that most will fully recover within the first week of the procedure and that there is no difference in expected recovery time according to whether the procedure is undertaken with local anaesthesia or conscious sedation.

**What is new?:**

The short-term recovery after endometrial ablation has been mapped in this trial. This information can be used in counselling women with heavy menstrual bleeding.

## Introduction

Endometrial ablation is a frequently used minimally invasive technique for the treatment of heavy menstrual bleeding. Over the years, many devices for endometrial ablation have been developed and evaluated. The NovaSure® system is a non-hysteroscopic technique using bipolar radiofrequency energy. This form of ablation achieves high amenorrhea and satisfaction rates (Rodriguez et al., 2019), making it an attractive option in the treatment of women with heavy menstrual bleeding. The maximum duration of the ablation is only 120 seconds, and therefore particularly suitable for outpatient treatment under local anaesthesia or conscious sedation (Rodriguez et al., 2019; Laberge et al., 2015; Reinders et al., 2017).

Most studies on the NovaSure® system investigate its effect on bleeding pattern, number of re- interventions, patient satisfaction and complication rates (Rodriguez et al., 2019). In contrast, up-to-date literature on short-term recovery and physical limitations that women may face is scarce. Most research on short-term recovery after endometrial ablation has been performed more than 10 years ago. In the meantime, many adjustments in hysteroscopic surgery have been made and the procedures have become less invasive (e.g., smaller devices, faster treatment, changes in anaesthesia and pain management). These innovations made it possible to perform this kind of treatment in an outpatient setting (Rodriguez et al., 2019; Clark et al., 2011; De Silva et al., 2021). The aim of this study is therefore to gain more insight into the short-term recovery after the NovaSure® endometrial ablation as it is performed nowadays. With this information, the pre-procedural counselling of women can be improved.

## Methods

### Study design, setting and population

This observational, prospective, and descriptive study was performed in Máxima MC, a teaching hospital in the Netherlands, between March 2019 and November 2021. The Medical Ethics Committee of the hospital confirmed that the Medical Research involving Human Subject Act (WMO) did not apply to the study. It is registered in the Netherlands Trial Register (NL8058). The reporting of this cohort study is in accordance with the ‘Strengthening the Reporting of Observational Studies in Epidemiology (STROBE)’ statement.

In Máxima MC, endometrial ablation is mainly performed using the NovaSure® device. As usual women were counselled about the available anaesthesia techniques: local anaesthesia (consisting of paracervical anaesthesia, and in the last year, in combination with intra-uterine fundal anaesthesia) and procedural sedation (combined with paracervical anaesthesia). General anaesthesia and/or spinal anaesthesia were offered only in exceptional cases (contra-indications for procedural sedation or the need of a concurrent laparoscopy).

These women were excluded from this study. All women, over 18 years of age and scheduled for a NovaSure® procedure under local anaesthesia or procedural sedation were eligible for this study. The exclusion criterion was poor understanding of the Dutch language. Eligible women received written information about the study and were informed by the gynaecologist. After counselling by a research assistant, all women who agreed to participate gave written informed consent prior to the procedure.

### Study procedure

**Figure 1:**
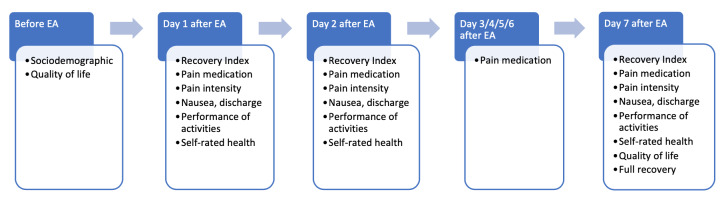
Time schedule and measured variables. EA: endometrial ablation.

A schematic overview of the study is shown in Figure 1. After signing informed consent, women completed the first questionnaire on sociodemographic data and health-related quality of life. All women were advised to use paracetamol 1000mg and naproxen 500mg one hour prior to the procedure. The NovaSure® procedure was performed. Postoperatively, women received instructions on how to prevent infection, but were not restricted in their physical activities. They were asked to complete questionnaires every day during the first week after the procedure. In addition, the gynaecologist completed a case report form (CRF) immediately after the procedure.

### Outcomes

The primary outcome was short-term recovery after the NovaSure® procedure, measured by the Recovery Index (RI-10) on day one, two and seven after ablation. The RI-10 is a ten-item questionnaire measuring postoperative recovery on five-point Likert scales ranging from full disagreement (1) to full agreement (5). The total score ranges from 10 to 50, where 50 indicates full recovery. Most of the items in the RI-10 refer to the postoperative situation, hence there is no baseline measurement available (Kluivers et al., 2008).

Secondary outcomes were health-related quality of life, use of analgesics, pain intensity, nausea, vaginal discharge (blood or fluid), ability to perform activities (light and heavy domestic chores, sports, work), self-reported health and feeling of full recovery in the first week after the treatment, all of which were assessed using patient questionnaires. Health-related quality of life was measured prior and seven days after the ablation with the EQ-5D-5L. The EQ-5D-5L measures health- related quality of life on five dimensions of health: mobility, self-care, usual activities, pain/discomfort, and anxiety/depression. The outcome measure is an index value between 0 and 1, with a higher value indicating a higher quality of life (EuroQol Research Foundation, 2019). Women were asked to note their used pain medication (type, dosage, amount) daily (day 1-7). On day one, two and seven the women had to fill in the actual pain intensity on the validated Visual Analogue Scale (VAS), with 0 indicating no pain and 10 indicating worst pain imaginable. Besides, the presence of nausea (yes/ no) and vaginal discharge (yes/no), the capability of performing work (yes/no), sporting activities (yes/ no) and domestic chores (Likert scale) and self- related health were asked in questionnaires on day one, two and seven. A five-point Likert scale was used to chart whether one could perform household tasks (full agreement (1) – full disagreement (5)). These household activities were divided into light and heavy tasks. Self-rated health was scored on a vertical Visual Analogue Scale (EQ-VAS). The EQ- VAS gives a value between 0 and 100, with 100 indicating the best of health imaginable (EuroQol Research Foundation, 2019). Finally, on day seven the women were asked how many days after the procedure they felt recovered, able to return to work and participate in sports.

The following sociodemographic characteristics were collected at baseline using a patient questionnaire: age, education level, living situation, daily work situation with level of physical demands. Furthermore, a CRF was completed on peri-operative items: use of preprocedural pain medication, anaesthetic technique, and abnormalities during the admission. Complications which arose during the procedure or during the first weeks in the postoperative were noted.

### Sample size and statistical analysis

It was decided in a discussion with experts that a total of 60 women, divided over both anaesthesia groups, should be included. The justification for this sample size is based on rationale about feasibility and literature about pilot and feasibility trials (Billingham et al., 2013). Only women who returned all questionnaires (baseline and follow-up) were included in the analysis. Descriptive statistics were presented for all included women together. Besides, the data were presented separately for women who received local anaesthesia and those who received procedural sedation. Continuous data were presented as mean and standard deviation (SD), or as median and interquartile range (IQR) in case of non-normal distribution. Nominal data were presented in numbers and percentages. Women who mentioned that they were not fully recovered after the first week and reported no recovery, thereafter, were taken into account in the measurement of the median recovery time. In case >25% of the women were not recovered after seven days, it was not possible to calculate the upper limit of the interquartile range (75th percentile). Then, the upper limit was described as >7 days. The same applies for resuming work and sports. Statistical analyses were performed using the software Statistical Package for the Social Sciences (SPSS), version 28 (IBM Corp, New York, USA).

## Results

### Participants

**Figure 2:**
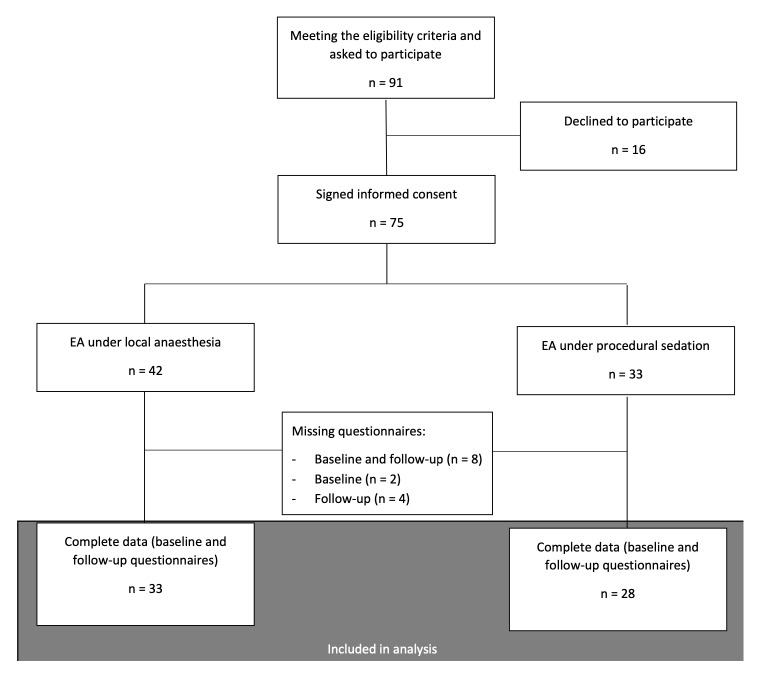
Flowchart

Between March 2019 and November 2021, 91 women were asked to participate in this study (Figure 2). Of these, 75 women agreed to participate and signed informed consent. These women underwent endometrial ablation with the anaesthesia technique of their preference (42 local anaesthesia, 33 procedural sedation). 61 women returned all questionnaires (baseline and follow-up questionnaires). These women were included in analysis.

### Baseline data

Baseline characteristics are shown in Table I. These are presented for the total group and both subgroups (local anaesthesia versus procedural sedation). The baseline characteristics of both subgroups were largely comparable. However, women who received local anaesthesia more commonly had a person at home who needed care (57.6% versus 28.6%).

**Table I:** Baseline characteristics.

	Total (n = 61)	Local anaesthesia (n = 33)	Procedural sedation (n = 28)
Age (years)^a^	44.4 (SD 5.7)	44.3 (SD 5.7)	44.5 (SD 5.7)
Living situation^b^ (1 missing)			
Living alone	11 (18.3%)	7 (21.2%)	4 (14.8%)
Living together with partner	49 (81.7%)	26 (78.8%)	23 (85.2%)
Dependent person at home (child/adult/caregiving)^b^			
Yes	27 (44.3%)	19 (57.6%)	8 (28.6%)
No	34 (55.7%)	14 (42.4%)	20 (71.4%)
Highest level of education^b^			
Primary education	1 (1.6%)	0	1 (3.6%)
Secondary education	5 (8.2%)	3 (9.1%)	2 (7.1%)
Senior vocational education	34 (55.7%)	19 (57.6%)	15 (53.6%)
Higher vocational education	18 (29.5%)	9 (27.3%)	9 (32.1%)
University	3 (4.9%)	2 (6.1%)	1 (3.6%)
Daily work situation (main job)^b^			
Paid job/entrepreneur	50 (82.0%)	27 (81.8%)	23 (82.1%)
Domestic work	9 (14.8%)	4 (12.1%)	5 (17.9%)
Unemployed	1 (1.6%)	1 (3.0%)	0
Charity	1 (1.6%)	1 (3.0%)	0
Paid and unpaid working hours per week^a^ (8 missing)	29.8 (SD 10.1)	28.5 (SD 9.3)	31.3 (SD 11.0)
Physical severity of work^b,d^ (2 missing)			
Light	24 (40.7%)	10 (31.3%)	14 (51.9%)
Medium	22 (37.3%)	14 (43.8%)	8 (29.6%)
Heavy	13 (22.0%)	8 (25.0%)	5 (18.5%)
EQ-5D-5L indexc	0.87 (0.81 – 1.00)	0.87 (0.81 – 1.00)	0.96 (0.78 – 1.00)

^a^Mean and standard deviation; ^b^Absolute number and percentage; ^c^Median and interquartile range ; ^d^Physical severity of work is defined as light (an office job), medium (working in a store with alternating lifting/sitting), heavy (most of the day physically active, e.g., construction worker or nurse.

### Procedural information

All endometrial ablation procedures were completed. Eight women did not use analgesics prior to the treatment (three local anaesthesia; five procedural sedation), 43 women used paracetamol and/or a non- steroidal anti-inflammatory drug, only one woman used a non-steroidal anti-inflammatory drug in combination with tramadol. The use of pain medication was not registered in nine women. No complications during the procedures were reported. One woman who received procedural sedation reported a vasovagal reaction after the treatment and another woman from the same group was hospitalized for an extended period (approximately four hours) because of pain. In the first week after the procedure, two women who received local anaesthesia were diagnosed with a genital tract infection and were treated with intravenous antibiotics in the hospital. Of the women who received procedural sedation, no infection was reported in the first week.

### Primary outcome: Recovery Index (RI-10)

**Figure 3:**
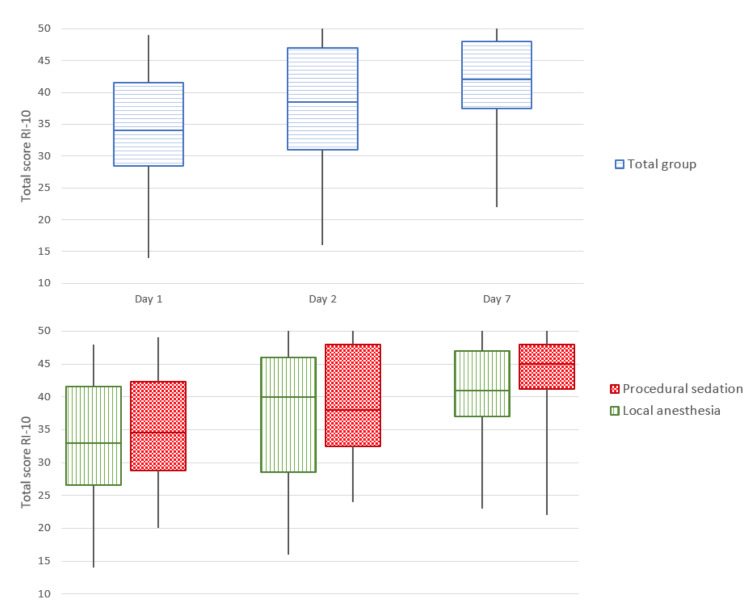
Recovery Index (RI-10).

The RI-10 was available for all women on day one and seven. For day two, there were seven missing values (four local anaesthesia; three procedural sedation). Figure 3 shows that the RI-10 increased during the first week with a median of 34 on day one (IQR 28.5-41.5), a median of 38.5 (IQR 31-47) on day two, and a median of 42 (IQR 37.5-48) at the end of the week. These values were comparable for women who received local anaesthesia and those who received procedural sedation.

### Secondary outcomes

#### Health-related quality of life (EQ-5D-5L)

The median index value of all included women before the NovaSure® procedure was 0.87 (IQR 0.81-1.00) (Table I). One week after the procedure, the median index value was 1.0 (IQR 0.84-1.00). For women who received local anaesthesia and procedural sedation this value was 0.87 (0.84-1.00) and 1.00 (0.84 – 1.00) respectively.

#### Pain scores, analgesia, and specific complaints

**Figure 4:**
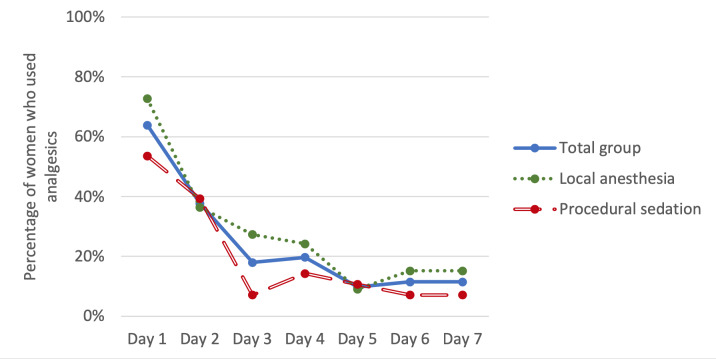
Use of analgesics.

Pain scores were missing for seven women on day two (four local anaesthesia; three procedural sedation) and for one woman on day seven (local sedation). The used pain medication was available for all women on every day. The pain scores decreased during the week after the procedure (Table II). In parallel, the use of analgesics decreased towards the end of the week (Figure 4). One day after the procedure, 63.9% of all women used pain medication (72.7% in the local anaesthesia group and 53.6% in the procedural sedation group). Seven days after the procedure, 11.5% of the women still used analgesics (15.2% in the local anaesthesia group and 7.1% in de procedural sedation group). The used analgesics were mainly paracetamol and/or NSAIDs. Only on the first day after the procedure one woman (procedural sedation) needed additional pain medication (tramadol). On the following days, there was no need for opioids or other pain medication.

**Table II:** Pain scores.

	Total	Local anaesthesia	Procedural sedation
Pain score day 1	2.0 (0 – 4.0)	2.0 (0 – 4.8)	2.0 (1.0 – 3.8)
Pain score day 2	0.85 (0 – 2.5)	0.0 (0 – 2.7)	1.0 (0 – 2.5)
Pain score day 7	0 (0 – 1)	0 (0 – 1.3)	0 (0 – 1)

Pain scores are reported as median and interquartile range.

Nausea was reported by 29.5% of the women (30.3% in the local anaesthesia group and 28.6% in the procedural sedation group). Most of these women only experienced nausea at the day of the procedure. In both groups, three women experienced nausea in the days after the procedure.

Only three women reported that they had no vaginal discharge at all in the first week after the procedure. 78.7% of all women reported that they still had discharge at the end of the first week, which was comparable in both groups (78.8% in the local anaesthesia group and 78.6% in the procedural sedation group). Only eight women reported it as bothersome, but none received further treatment.

#### Daily activities

**Figure 5:**
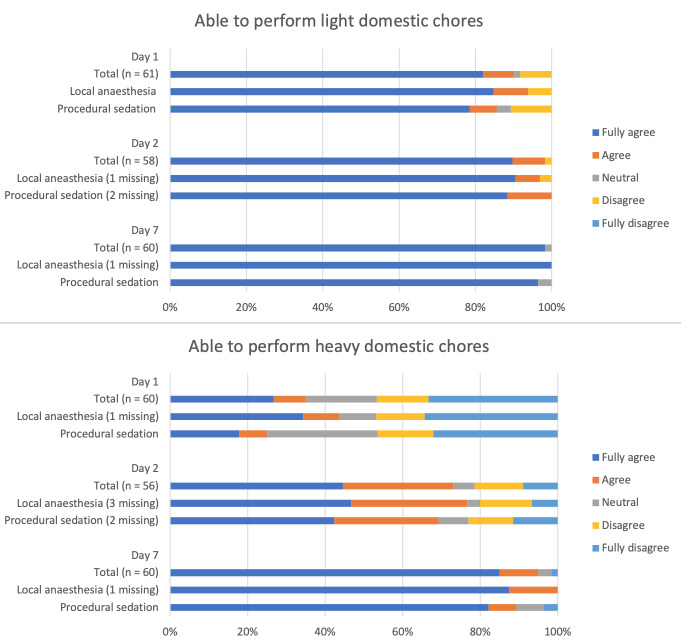
Performing domestic chores.

The results on performing domestic chores are presented in Figure 5. The median time it took women to return to work, resume sporting activities and feel completely recovered was reported in Table III.

**Table III:** Daily activities.

	Total	Local anaesthesia	Procedural sedation
Return to work (days) (n = 61)	2.0 (1.5 – 4.5)	2.0 (1.0 – 5.0)	2.5 (2.0 – 4.5)
Resume sporting activities (days) (n = 40)	5.5 (3.0 – 7.5)	5.0 (2.5 – >7)^a^	6.0 (3.0 – 7.0)
Feel completely recovered (days) (n = 61)	5.0 (2.0 – 7.00)	5.0 (2.0 – >7)^a^	4.0 (2.0 – 7.0)

The results are presented as median and interquartile range.

^a^Due to missing data (not resumed sporting activities or recovered within 7 days) the 75^th^ percentile was not reached. At least this value is >7 days.

On day two after treatment, 57.4% of the women were able to return work. After seven days, five women were not able to return to work (four local anaesthesia; one procedural sedation). Forty women indicated that they practice sports (20 women in both groups). Of these, 22.5% were able to perform sporting activities two days after treatment. After seven days, 10 of those 40 women were still not able to resume sporting activities (six local anaesthesia; four procedural sedation). Of the total cohort, 23% of the women indicated not feeling fully recovered after seven days (nine local anaesthesia; five procedural sedation). Of these women, two women mentioned another timepoint that they felt fully recovered (>7 days). Among the women who felt not fully recovered after two days were the two women with infection postoperatively.

#### Self-reporting health (EQ-VAS)

The EQ-VAS was available for all included women on days one and seven. For day two, there were seven missing values (four local anaesthesia; three procedural sedation). The EQ-VAS increased during the first week after the procedure. The median scores of the total group were: 70.0 (57.5- 80.0) on day 1, 80.0 (60.0-90.0) on day 2, 90.0 (80.0-96.5) on day 7. The scores were comparable in both groups (local anaesthesia: 65.0 (52.5 – 80.0), 75.0 (55.0 – 90.0) and 87.0 (80-97.5); procedural sedation: 70.0 (60.0 – 80.0), 80.0 (65.0 – 90.0) and 90.0 (80.0 – 97.25)).

## Discussion

The aim of this cohort study was to gain more insight into the short-term recovery after NovaSure® endometrial ablation to improve the pre-procedural counselling of patients. The primary outcome, the RI-10, showed an upward trend in recovery during the first week after the procedure. The median time that it takes women to feel fully recovered after the procedure was five days. The median time in which women were able to return to their work was two days and resuming sporting activities was 5.5 days. 90% of the women were able to perform light domestic chores on the first day after the procedure. While almost 75% of the women were able to perform heavy domestic chores after two days. Most women only needed pain medication on the first day after the procedure. Vaginal discharge (blood and fluid) was a side-effect that almost every woman experienced after NovaSure®. After seven days, 23% of the women indicated that they were not fully recovered. There was only a small group of women that still needed pain medication and were not able to resume normal activities yet. There was no remarkable difference in recovery between the women who underwent the procedure under local anaesthesia compared to those who received procedural sedation.

Important strengths of this study are its prospective design and uniform comprehensive data collection. Moreover, by asking real-time information with a questionnaire daily, recall bias is minimised, and a precise overview of the recovery time is made.

This study also has some limitations. First, no sample size calculation was performed to analyse differences between women who received local anaesthesia and those who received procedural sedation. The primary goal of this trial was to gain more insight in the recovery after endometrial ablation overall, instead of comparing those groups. No relevant differences in recovery were found between both groups, but it is possible that the groups were too small to detect a difference. Second, the two subgroups were not created by randomisation which could have led to selection bias. Nevertheless, the group characteristics at baseline were largely comparable. Besides, it is to be expected that women are not willing to be randomised because of a strong preference for one or the other anaesthetic technique. Lastly, there is a risk of non-response bias. The proportion of patients lost to follow-up is 6% (four women did not return the follow-up questionnaires after they filled out the baseline questionnaire).

Most studies investigating endometrial ablation focus on its long-term effects (e.g., efficacy on heavy menstrual bleeding, quality of life and re-surgery) and were not designed to describe the short-term recovery. The authors of a recent Cochrane review (Rodriguez et al., 2021) comparing endometrial ablation with hysterectomy have come to the expected conclusion that a hysterectomy is more effective in resolving bleeding problems with higher satisfaction rates. On the other hand, hospital stay, and recovery time were shorter after endometrial ablation. Most of the studies included in the analysis on recovery were older than 15 years, except for the HEALTH trial (Cooper et al., 2019), which was published while our study was being conducted. In the HEALTH trial, endometrial ablation and laparoscopic supracervical hysterectomy were compared. The ablation techniques used were thermal balloon and bipolar radiofrequency. Of the 297 women who underwent endometrial ablation, 5% stayed in the hospital for more than 24 hours, 38% needed opiates postoperatively, and the median time of return to work, sport and social activities was longer than we found in our study (paid work ten days; unpaid work seven days; sporting or social activities 14 days). We could only speculate why the recovery after ablation took longer in the trial of Cooper et al. (2019). Relevant differences with our study were the high rate of general anaesthesia (95%) and the higher change of recall bias because women had to answer questions six weeks after surgery. The postoperative instructions given to the included women were not described in the article. In our trial, women were told that there were no restrictions in physical activity. This may influence the time that activities were resumed. Earlier studies (published >10 years ago) reported more similar results on resuming activities compared to our study (Brun et al., 2006; Clark et al., 2011; Marsh et al., 2005; Milligan et al., 2002; Pellicano et al., 2002; Prasad and Powell, 2008; Wallage et al., 2003). For returning to normal activities the documentation of data was heterogeneous, but all studies reported that most women resumed normal activities within the first week, as in our study. Most women returned to their job within 5-7 days, compared to two days in our study. Therefore, it seems that the time women were off from work has become shorter over the last decade. Probably, this is due to multiple developments in the field of minimally invasive surgery. There were, for example, developments related to the ablation technique itself (smaller devices, shorter ablation time etc.), the anaesthetic techniques (procedural sedation and more enhanced local anaesthesia) and to postoperative care (shorter hospital stay, different postoperative instructions).

Women with heavy menstrual bleeding should be adequately counselled about treatment options. This counselling should include the type of treatment and the expected effect. Besides that, the expected recovery from surgery and the personal situation and wishes of each unique patient should be discussed, to help her make an informed choice. Regarding Novasure® endometrial ablation, the doctor can tell women to take a recovery time of five days into consideration, with the majority resuming work already after two days. Mostly, pain medication is only necessary on the first two days.

In conclusion, this observational study provides useful data on the short-term recovery after NovaSure® endometrial ablation. With this information, women suffering from heavy menstrual bleeding can be counselled for endometrial ablation with specific information about the expected recovery in the first seven days after the procedure. This makes it possible for patients to make a better-informed decision for treatment.
